# Work-Related Stress and Coping: A Comparative Analysis of On-Site and Office-Based Workers in UK Building Construction

**DOI:** 10.3390/healthcare12212117

**Published:** 2024-10-24

**Authors:** Rachel Blair Winkler, Campbell Middleton, Olivia Remes

**Affiliations:** 1 Saïd Business School, University of Oxford, Oxford OX1 2JD, UK; rachel.winkler@pmb.ox.ac.uk; 2 Department of Engineering, Laing O’Rourke Centre for Construction Engineering and Technology, University of Cambridge, Cambridge CB3 0FS, UK

**Keywords:** construction industry, workplace stress, coping mechanisms, stress management strategies, mental health, wellbeing, UK building construction, qualitative research in construction

## Abstract

Background: There are increasing mental health concerns in the construction industry workforce, with stress being a primary concern. This qualitative study investigates stress experiences and the management of stress in office-based and on-site workers in the UK building construction sector. This study can provide key insights for construction, but also potentially other industries which struggle with high stress levels among their employees. Methods: A total of 40 semi-structured interviews (20 on-site/20 office-based) were conducted at large-scale building construction projects in the southeast UK. Framework method analysis was used to derive an understanding of stress in the workplace and coping strategies. Results: The study identified two major themes: the negative influence of work stress on personal and professional wellbeing, and the management of stress through proactive and reactive coping strategies. Results indicated that on-site workers frequently cited high workloads, tight deadlines, and safety concerns, whereas office-based workers tended to highlight job complexity and organisational pressures. Both population sub-groups reported stress affecting their work performance, with site-workers having the added concern of physical health and safety. Coping strategies among workers tended to rely on support networks, outside-the-workplace hobbies, and boundary-setting, among others. Conclusions: Stress remains a significant problem in the workplace and affects wellbeing; however, there are ways to mitigate the stress. Our findings could provide a foundation for intervention development.

## 1. Introduction

Poor mental health in construction is a significant challenge. This is something that is often not discussed, and there is insufficient public awareness regarding this issue. The construction industry experiences substantial workplace challenges that affect its workers. Some of these can include work-related hazards, unstable jobs, and temporary contracts [1,2]. Also, stressors in the industry are related to long hours and insufficient support in the workplace, and in order to deal with problems, some workers use ineffective coping (i.e., substance use and self-blame) [3].

A key underlying mechanism linked to poor mental health in construction is high stress. In fact, the construction industry is one of the most stressful industries to be part of. According to a survey by the Chartered Institute of Building (CIOB) [4] conducted on 847 construction industry professionals, almost 70% of participants experienced issues such as stress, anxiety, or depression. This was directly linked to being employed in construction. Occupational stress among participants in the research by CIOB appeared to stem from issues, such as job demands and insufficient social support in the workplace (including communication problems). While the extant evidence base on wellbeing and mental health in construction has provided valuable insights, not enough work has been conducted on stress and the wide range of factors that are linked to this experience. A systematic review [3] conducted in 2023 shed light on psychological-related factors and demands in construction, and it showed that stress is linked to high workload and aspects concerned with safety, among other variables. Further information on specific experiences of stress in construction is needed: for example, the impact it may have on one’s work life and personal life, and the ways that construction professionals cope with it. Work on stress in the context of construction is important for a number of reasons.

First, stress is a key risk factor for physical ill health and psychopathology. Stress, for example, can have an impact on the nervous, immune, and neuroendocrine systems [5], and it has been linked to telomere damage [6], as well as accelerated aging processes [7,8]. When people experience chronic stress, this can also increase the risk of having anxiety, depression, and related challenges [3,9]. Thus, given the negative impact of ongoing stress, and the fact that the construction industry employs millions of people (who may be at high risk of stress), it is important to adequately assess psychological experiences in this sector. As millions of new workers will be needed by this industry over the coming years, more research is needed to better understand the experiences of stress in construction. This can inform targeted prevention and intervention strategies.

While stress levels in construction have been examined in the literature, important gaps remain. The experiences of stress (along with coping strategies) in the context of different workplace environments (site versus office settings) have not been sufficiently explored. Also, there is limited knowledge on the impact of the workplace environment on individuals in construction [10] (especially as it relates to the theme of stress and wellbeing), and related perceptions of those working in construction. As such, this study seeks to address these gaps.

### 1.1. Theoretical Framework and Research Aims

Our study on stress in construction is guided by Lazarus and Folkman’s Transactional Model of Stress and Coping [11]. This model was chosen because it provides a framework for understanding how individuals perceive and respond to stress, which is especially useful for interpreting stress in the workplace. This model establishes that stress arises from the interaction between an individual and the environment; it further explains how individuals appraise stressors and subsequently employ coping strategies.

This theoretical foundation guides the development of the research methods, instruments, and data interpretation. In applying this framework, our study aims to elucidate the relationship between stressors, individual appraisals of stress, and coping strategies among on-site and office-based building construction workers.

### 1.2. Research Objectives

While understanding stress specific to each of these population sub-groups can help tailor specific interventions, it may also be illuminating to study stress and the management of stress similarly across office-based and on-site workers. The UK provides an interesting opportunity to explore stress and its impact on aspects of wellbeing. The construction sector is a good example of public/private partnership. In recent years, the UK government announced ambitious aims for sustainability, economic development, and supporting workforce mental health (and more generally, health and safety) in partnership with this industry [12]. The rationale for our research is that insights can be gained at the cutting edge beyond current practice by examining a comparatively forward-thinking sector. This, in turn, may provide a perspective that others may use for guidance.

To address the gaps in the research base, this paper is a qualitative study comparing workplace stress in on-site and office-based workers in UK building construction. This research seeks to understand the following:

1. The levels of stress experienced by on-site and office-based workers.
2. The primary sources of stress for these two groups.
3. The coping strategies employed by on-site and office-based workers.
4. How stress and coping mechanisms impact the wellbeing of workers.

By addressing these objectives, this study aims to improve the understanding of stress specific to the industry context. Providing better insight into the manifestation and mitigation of stress, with perspectives from different population sub-groups in building construction, may ultimately help improve wellbeing in the industry.

## 2. Materials and Methods

### 2.1. Participants

The study was designed with two population sub-groups—site and office—to identify themes that are both industry-wide and specific to each population sub-group. The study was populated to obtain equal representation from both sub-groups and allow for comparative analysis. Criteria for inclusion were a minimum of 50% of work hours spent on-site or in-office (to characterise the population sub-group) and direct management of people or operations on an active building construction project. Participants were informed they were participating in a voluntary and confidential study of stress and wellbeing in construction lasting no more than 45 min. They were recruited by the authors on-site at random.

The study included 40 participants (33 males and 7 females) from 12 different employers across 4 building sites. Among the participants, 19 were speciality subcontractors, 16 were general contractors, and 5 were consultants. The roles ranged from site management to mechanical, electrical, and plumbing subcontractors. The majority of participants (28) were from the UK, while 12 were not. Additionally, 26 participants had dependants, and 14 did not.

### 2.2. Instruments

The instruments used in this study include a demographic questionnaire and a semi-structured interview guide. The demographic questionnaire collected basic participant information, summarised in Section 2.1. The authors developed the remaining questions based on input from domain experts and existing construction industry-specific literature. The overarching structure was informed by the Lazarus and Folkman model, [11] separating a discussion of stress and coping in two parts of the interview. The structured questions were guided by frequently-referenced industry reports from the Construction Industry Training Board [13] and the Chartered Institute of Builders [4], and derived from Wu and Yao’s (2018) academic study [14] on the development of a job stress scale for construction industry. Our research—and in particular, the topic of stress—were also guided by a more recent CIOB survey [15] on over 2000 respondents. This survey showed that 97% of individuals in the construction industry experienced stress in the previous 12 months [15]. As a result, we decided to explore this dimension further through our own research.

Structured questions in our study were followed by an unstructured opportunity for open-ended reflections on stress and coping in the workplace.

The rationale for using the methods described was to capture the nuanced and contextual data necessary for understanding the experiences of construction workers. The qualitative methods and detailed questions used in this research provide deeper insights into the personal and situational factors influencing stress and the management of stress [16,17]. This approach allows for the flexibility to explore individual experiences in detail, which is necessary to complete a holistic understanding of stress and coping in the industry.

### 2.3. Data Collection Procedures

Four building construction projects in the southeastern UK were selected for this study due to their scope and scale and representation of large and complex building construction projects in the UK. Construction industry stakeholders facilitated the site visits. All interviews were in person and recorded on Microsoft Teams for subsequent analysis. Interviews were conducted at each of the building construction sites in May and June 2023 over consecutive two-day intervals. Interviews continued until recurrent themes emerged from both population sub-groups, and equal numbers of on-site and office-based participants were interviewed (namely, 20 respondents in each sub-group).

Demographic questions were initially asked, followed by structured interview questions where respondents selected options from a printed sheet. Subsequently, respondents were asked additional open-ended questions to explore their stress and coping experiences in more detail. In sum, 1140 min of interview material was collected, ensuring comprehensive data collection. The mean interview duration of participants classified as office-based workers was 32.5 min, and the mean interview duration of on-site workers was 32.6 min.

Ethics approval was obtained from the University of Cambridge before the commencement of the study. Participants were asked for consent to record the interviews, with recordings used solely for transcription and analysis. Each participant was assigned an identification number to anonymise the data. All participants reviewed an information sheet identifying their rights and the researcher’s responsibilities, and signed consent forms were collected in accordance with university policies.

### 2.4. Data Analysis

First, descriptive analysis was conducted on the data from structured questions on stress levels, sources of stress, and coping strategies in pursuit of research objectives 1, 2, and 3 (Section 1.2). Data are summarised by frequency, compiled separately by population sub-group (on-site vs. office-based) and in total. The descriptive analysis provides a baseline of stress and coping and creates a context for themes to be developed and explored in the next phase of analysis.

Second, and to address research objective 4 (Section 1.2), an inductive, qualitative content analysis was carried out [16]. Specifically, framework method analysis was employed to derive themes and generate a comparison between population sub-groups [18]. This process involved multiple stages, including data familiarisation, coding, theme development, charting, mapping, and interpretation, retaining links to the data at each phase. The framework method was chosen for this study because it facilitates an overarching, holistic, and comparative view of stress and coping experiences among all participants, as well as those specific to on-site or office-based workers.

The analysis resulted in frameworks (e.g., ‘main themes’). Each framework was composed of categories (e.g., ‘subthemes’). Each framework is also a matrix composed of a theme, subthemes, the study’s population sub-groups (site and office), and a measure of subtheme strength. The subtheme strength is indicated in the framework matrix by the number of participants (by site and office) who raised a topic mapped to a category in the framework.

## 3. Results

*Brief overview of the section*

The results from the descriptive analysis and the framework analysis are presented.

First, the descriptive analysis explores characterisations of work stress (perceived levels of stress, sources of stress, and the management of stress), presenting results separately by population sub-group with a combined count indicated in each table. Next, themes and subthemes from the framework analysis are presented. The two main frameworks generated from this analysis are (1) the negative impact of work stress on personal and professional wellbeing and (2) coping mechanisms to manage work stress (this is contained in the second part of the ‘Results’).

### 3.1. Characterisations of Work Stress

#### 3.1.1. Stress Levels

To establish a baseline picture of stress, all participants were asked to characterise levels of work stress. Participants were asked the following question with a five-point response format, “*In general, how stressful do you find your job?*” The scale ranged from (1) *not stressful at all* to (5) *extremely stressful*. Table 1 summarises self-reported stress by sub-group, indicating 70% of site workers and 85% of office workers find their jobs *moderately stressful*, *very stressful*, or *extremely stressful*. No participants characterised their jobs as *not stressful*.

#### 3.1.2. Sources of Stress

To illuminate underlying issues, this study also explored sources of stress. Participants were asked to identify stressors (as yes/no) from a predefined list. They were also given the opportunity to identify additional stressors that might not have been captured by the list. Table 2 contains frequency counts of various sources of stress, and volunteered observations regarding any additional stressors are elaborated in-text below.

The most frequently-identified sources of stress were *high workloads* (32 participants), *tight deadlines* (30 participants), and *situations not under one’s control* (24 participants). Site workers more frequently identified *situations not under one’s control*, *responsibility for the safety of others*, and *having a dangerous job*. Office workers more frequently cited *overtime* and *job instability*. Outside the pre-defined list, over 50% of on-site workers elaborated on sources of stress, which can be categorised as the following: (1) *last-minute changes* and (2) *lack of communication*. Office-based workers added the following sources of stress with similar frequency: (1) *managing complexity* and (2) *stress from personal/family issues*.

#### 3.1.3. Coping Mechanisms

To understand the management of work stress at the highest level, participants were asked to give a yes/no response to a list of coping mechanisms, summarised in Table 3. Results showed that about two-thirds of all participants would cope with stress by telling someone about it. The most frequent elaboration from site workers was ‘*family*’ or ‘*a friend*’ as trusted confidants to talk to about workplace stress. The most frequent elaboration from office workers was ‘*a colleague*’ to talk to during a stressful time. Potentially related to ineffective coping, 21/40 participants cited *trying to block* stress or *keeping it to oneself*. Less frequently overall, but identified in 50% of office workers was *solutionising*.

In sum, findings thus far show that 100% of participants experience some level of job stress from various sources, and participants have different ways of coping with stress. The following framework analysis highlights the negative influence of work stress on professional and personal wellbeing, and how participants manage work stress.

### 3.2. The Negative Influence of Stress on Personal and Professional Wellbeing

Framework 1, enumerated in Table 4, further defines the ways work stress was found to influence various aspects related to wellbeing. A total of 218 sections of textual data mapped to this framework, and explore how stress impacts aspects of participants’ wellbeing. Subthemes are labelled as *professional factors* or *personal factors* depending on how the influence of stress was described by participants. At the highest level, 80% of participants signalled that work stress can negatively impact their jobs, and 75% indicated moderate stress can negatively impact their personal/family lives.

Subthemes on the negative impact of stress can broadly be categorised as *professional*; this relates to the negative influence of stress on the quality or quantity of work performance, and the management of personal or others’ safety. Stress has a negative influence on all the aforementioned factors. Office workers noted a decrease in work performance more often (compared to site workers); further, feelings of anxiety were also mentioned in relation to performance. Site workers noted an impact (of stress) related to safety more frequently; the word ‘distraction’ was used in the context of a higher-stakes environment.

*“If it (work) all piles up, I get so anxious that I can’t do anything. I just spin.”*

*(office)*

*“It’s not a good idea to get on a 200-foot crane when you’re having a bad day… you can’t be distracted up there.”*

*(site)*

Subthemes on stress that can be detrimental to *personal* wellbeing are influences on (physical) health or personal/family life. In total, 65% of on-site and office-based workers indicated work stress affects health or habits related to health, such as loss of sleep, changes in routines, or physical pain, like muscle tension. Site workers noted more than office (70% vs. 55%) that work stress would negatively impact their home life. ‘Withdrawal’ or a ‘short temper’ with immediate family members were called out.

*“I’m normally keen to play football… When it’s real stressful, I can’t find the energy.”*

*(office)*

*“The first thing to go is sleep. It’s always the first.”*

*(site)*

*“Sometimes I’m a bit short-tempered with my wife and kids. They notice.”*

*(site)*

These results illuminate how work stress can have a detrimental impact on personal or professional wellbeing in both sub-groups of the study.

### 3.3. Coping Mechanisms to Manage Work Stress

Framework 2, outlined in Table 5, is on the management of work stress, with categories reflecting individual coping strategies. A total of 191 pieces of textual data related to how participants manage stress were the building blocks of this framework. Framework 2 illuminates coping strategies that can fall into two categories of stress management: *reactive coping* or *proactive coping*. Most participants (39/40) described coping mechanisms they adopted to tackle stress. These comments fell into four categories: support networks, hobbies, habits, and mindset. Some participants mentioned more than one coping strategy (e.g., relying on support networks and hobbies).

Reactive coping mechanisms refer to strategies employed after the onset of stress. Participants remarked on support networks of family and friends outside work, describing these networks as regulating, using terms like ‘provides perspective’ and ’lets the pressure out’. Hobbies were also mentioned, with participants describing personal activities ranging from sports to art. However, when work gets really busy, sometimes hobbies (the ‘stress-relievers’) fall to the wayside. These two subthemes—support networks and hobbies—were identified most by site workers.

*“My wife knows when I walk through the door if I’ve had a bad day. We usually talk about it, then I can move on and play with my boys, which helps me reset.”*

*(site)*

Proactive coping mechanisms, on the other hand, are strategies employed to prevent the onset of stress on a pre-emptive and ongoing basis. These include developing healthy habits and maintaining a positive mindset and were emphasised more by office workers. Healthy habits were described as an opportunity for preventing stress, with activities like time in nature, exercise, and meditation. Other habits mentioned were boundary-setting, both digital and physical, between work and home. Lastly, the concept of mindset emerged. Some office workers (11/20) mentioned a positive frame of reference as important given the stress inherent to construction. Over 50% of office workers described healthy habits or mindset changes as proactive coping mechanisms against stress, with comments like,

*“I use the app… to help with meditation and I exercise… I find I have to do both every day to feel OK.”*

*(office)*

*“When I go home, I turn my phone off and digitally disconnect. It can wait until tomorrow.”*

*(office)*

## 4. Discussion

This study shows that employees in construction find their jobs highly stressful. In particular, 70% of site workers and 85% of office workers find their jobs as moderately stressful, very stressful, or extremely stressful. This is in line with the extant evidence base, which shows that stress is a common experience in construction. In fact, research by CIOB similarly shows that about 70% of construction employees experience stress (among a number of other issues) [4]. While the extant evidence base has looked into the levels of stress in construction, there are gaps in the literature with respect to the experience of site- versus office-based workers. These two population sub-groups tend to be exposed to different work environments, and, as such, need to be analysed separately. In this study, results show that stress levels indeed vary between these two sub-groups.

In this research, the most common sources of stress found in construction employees were: high workloads, tight deadlines, and situations not being under one’s control. When findings were compared for site- versus office-based workers, the former more often identified the following aspects as sources of stress: situations not being under one’s control; being responsible for the safety of others; and having a dangerous job. A reason for this could be that site workers tend to be exposed to hazards that can increase the risk of injury. This research also showed that office workers more often cited the following as sources of stress: overtime and job instability. Given that the workload in construction settings is high and there can be uncertainty when it comes to the duration of employment, such factors can add pressure and make it difficult to cope. Our research fills a gap in the literature, and shows the most common sources of stress that construction employees working on sites vs. offices experience. This can inform the development of tailored wellbeing initiatives.

In addition, our research shows that coping mechanisms for stress revolve around social support. In particular, site workers identified family and friends as being sources of help, while office-based workers indicated that they would consult with a colleague for potential problems. The literature has shown the importance that social support has when it comes to stress, and overall mental health. In fact, researchers from Yale University School of Medicine and Mount Sinai School of Medicine suggested that social support can help people bounce back from stress. An underlying mechanism for this could be due to the implication of the hypothalamic–pituitary–adrenal (HPA) axis (which is linked to stress management), among other factors [19].

### Future Research

Future research should repeat this study using tools such as ‘The Perceived Stress Scale’ [20,21] to measure stress. The ‘Perceived Stress Scale’ is one of the most well-known scales for assessing perceived stress and includes items, such as ability to cope, feelings of control, feelings about difficulties/irritations, and ability to overcome challenges [20]. Using such a scale could provide insight into the degree of stress people feel. This scale shows whether respondents believe their lives or the factors going on their lives are unpredictable, uncontrollable, and overwhelming [21,22].

To gain further insight into the experiences of construction professionals, this study should also be repeated using the Depression, Anxiety, and Stress Scales (DASS)-21. This tool is well-known and robust [23,24], and contains a part focusing on stress.

In terms of coping scales, we recommend this research is also repeated using tools, such as The COPE; there are a number of frequently-used instruments as highlighted by Toyo University in Japan [25]. Using some of these measuring instruments could provide additional insight into our work on coping.

## 5. Conclusions

This study illuminates the experiences and differences of stress between on-site and office-based workers in the UK building construction sector. While both population sub-groups experience high stress levels, with 70% of on-site and 85% of office-based workers reporting moderate to extreme stress, the sources and impacts of stress differ. This study challenges what may be an oversimplification or oversight in existing research by highlighting distinct stressors and coping mechanisms for each population sub-group in a single comparative framework. The research calls for a more complex understanding of stress in the construction industry, moving beyond the traditional characterisation of on-site versus office-based stress.

The insights gained from this study advocate for a more inclusive approach to stress management. This research suggests programs to mitigate stress and promote wellbeing should take into account the different professional responsibilities, environmental conditions, and organisational cultures of different population sub-groups in construction. Future research could explore differences by job role in greater depth, with the aim of developing targeted intervention programs to improve overall worker wellbeing.

## Figures and Tables

**Table 1:** Response Format Stress Level.

Response	On-Site (n = 20)	Office-Based (n = 20)	Total Count (n = 40)
(1) Not stressful	0	0	0
(2) Somewhat stressful	6	3	9
(3) Moderately stressful	6	13	19
(4) Very stressful	8	3	11
(5) Extremely stressful	0	1	1

**Table 2:** Sources of Stress Indicated ‘Yes’ by Participants.

Statement	On-Site * (n = 20)	Office-Based * (n = 20)	Total Count (n = 40)
High workloads	16	16	32
Tight deadlines	14	16	30
Situations not under your control	15	9	24
Overtime	7	11	18
Responsible for safety of others	13	5	18
Dangerous job	10	0	10
Personal financial concerns	5	7	12
Relationships at work	5	6	11
Manager relationship	4	5	9
Unstable job	2	6	8
Commute time	1	3	4
Job/family clash	1	1	2

* 20 on-site and 20 office-based workers responded to the questions/statements. Participants tended to indicate ‘yes’ to more than one statement (source of stress).

**Table 3:** Coping Strategies.

Statement	On-Site * (n = 20)	Office-Based * (n = 20)	Total Count (n = 40)
I tell someone about it. (If so, who?)	14	13	27
I try to block it or keep it to myself.	9	12	21
I try to find ways to solve it.	6	10	16

* 20 on-site and 20 office-based workers responded to the questions/statements. Some participants indicated the use of more than one coping strategy.

**Table 4:** Framework 1. The negative influence of stress on ‘personal’ and ‘professional’ wellbeing.

	*Professional Factors*		*Personal Factors*	
Sub-Group	Work Performance	Safety	Health	Family Life
On-site * (n = 20)	11	10	13	14
Office-based * (n = 20)	16	2	13	11

* 20 on-site and 20 office-based workers were interviewed. The frameworks indicate the number of participants with data mapped to each sub-theme. Note that each participant is counted only once, even if a subtheme was mentioned multiple times.

**Table 5:** Framework 2. Coping mechanisms to manage work stress.

	*Reactive Coping*		*Proactive Coping*	
Sub-Group	Support Networks	Hobbies	Habits	Mindset
On-site * (n = 20)	17	15	10	7
Office-based * (n = 20)	11	12	15	11

* 20 on-site and 20 office-based workers were interviewed. The frameworks indicate the number of participants with data mapped to each sub-theme. Note that each participant is counted only once, even if a subtheme was mentioned multiple times.

## Data Availability

The original contributions presented in the study are included in the article.
